# CDK4/6 inhibition sensitizes MEK inhibition by inhibiting cell cycle and proliferation in pancreatic ductal adenocarcinoma

**DOI:** 10.1038/s41598-024-57417-z

**Published:** 2024-04-10

**Authors:** Ke Cheng, Zijian Zhou, Qiangxing Chen, Zixin Chen, Yu Cai, He Cai, Shangdi Wu, Pan Gao, Yunqiang Cai, Jin Zhou, Xin Wang, Zhong Wu, Bing Peng

**Affiliations:** 1 Division of Pancreatic Surgery, Department of General Surgery, West China Hospital of Sichuan University, Chengdu, China; 2 Division of Liver Surgery, Department of General Surgery, West China Hospital of Sichuan University, Chengdu, China

**Keywords:** PDAC, Trametinib, Palbociclib, Drug resistance, Cancer genetics, Cancer screening, Cancer therapy

## Abstract

Pancreatic ductal adenocarcinoma (PDAC) is not sensitive to most chemotherapy drugs, leading to poor chemotherapy efficacy. Recently, Trametinib and Palbociclib have promising prospects in the treatment of pancreatic cancer. This article aims to explore the effects of Trametinib on pancreatic cancer and address the underlying mechanism of resistance as well as its reversal strategies. The GDSC (Genomics of Drug Sensitivity in Cancer) and CTD2 (Cancer Target Discovery and Development) were utilized to screen the potential drug candidate in PDAC cell lines. The dose-increase method combined with the high-dose shock method was applied to induce the Trametinib-resistant PANC-1 and MIA PaCa-2 cell lines. The CCK8 proliferation assay, colony formation assay, flow cytometry, and western blot were conducted to verify the inhibitory effect of Trametinib and Palbociclib. RNA-seq was performed in resistant PDAC cell lines to find the differential expression genes related to drug resistance and predict pathways leading to the reversal of Trametinib resistance. The GDSC and CTD2 database screening revealed that Trametinib demonstrates a significant inhibitory effect on PDAC. We found that Trametinib has a lower IC_50_ than Gemcitabine in PDAC cell lines. Both Trametinib and Gemcitabine can decrease the proliferation capacity of pancreatic cells, induce cell cycle arrest, and increase apoptosis. Simultaneously, the phosphorylation of the AKT and ERK pathways were inhibited by the treatment of Trametinib. In addition, the RNA-seq of Trametinib-induced resistance PDAC cell lines reveals that the cyclin-dependent kinase (CDK)-RB-E2F regulatory axis and G2/M DNA damage checkpoint might lead the drug resistance. Besides, the combination of Trametinib with Palbociclib could inhibit the proliferation and cell cycle of both resistant cells lines and also restore the sensitivity of drug-resistant cells to Trametinib. Last but not least, the interferon-α and interferon-γ expression were upregulated in resistance cell lines, which might lead to the reversal of drug resistance. The study shows Trametinib has a critical inhibitory effect on PDAC. Besides, the combination of Trametinib with Palbociclib can inhibit the proliferation of PDAC-resistant cells.

## Introduction

Pancreatic ductal adenocarcinoma (PDAC) is a highly lethal malignancy with a poor prognosis. The five-year survival rate for PDAC remains significantly lower than that of various other solid tumors, such as breast, colon, and stomach cancers, with a rate of less than 10%, and it is predicted to escalate to the second-highest mortality rate by 2030^1^. Surgery is currently the best treatment option for PDAC. However, many cases have lost the opportunity for surgery when they are diagnosed^2^. Even if surgical treatment is possible, the recurrence and metastasis rates after surgery are high. Therefore, comprehensive treatment including chemotherapy and targeted therapy is important. However, PDAC is not sensitive to most chemotherapy drugs, leading to poor chemotherapy efficacy^3^.

Recently, researchers have been working hard to find effective targeted therapy sites in recent decades, among which the KRAS (Kirsten rat sarcoma viral oncogene homolog) protein is an important target. The activation of the MAPK (Mitogen-Activated Protein Kinase) pathway occurs in almost 90% of PDAC patients. However, previous studies have shown that the KRAS protein at the small molecule inhibitor level has not achieved the desired therapeutic effect due to severe side effects^1^. Therefore, researchers have focused on inhibiting other downstream of KRAS such as the MAPK pathway, PI3K/AKT (Phosphoinositide 3-kinase/Protein Kinase B) pathway, etc^4–6^. Recently, studies targeting MEK1/2 (Mitogen-Activated Protein Kinase Kinase) inhibition in the MAPK pathway have achieved some success.

The protagonist of this paper, Trametinib, is a selective inhibitor of MEK1/2, which can downregulate the phosphorylation level of downstream proteins such as ERK (Extracellular Signal-Regulated Kinase) by inhibiting MEK expression^7^. This drug is currently approved for use in combination with Dabrafenib for unresectable or metastatic melanoma, metastatic BRAFV600E mutation-positive non-small-cell lung cancers, and metastatic BRAFV600E mutation-positive anaplastic thyroid cancer. It also represents a new standard-of-care option for patients with recurrent low-grade serous carcinoma^8^. Besides, Palbociclib is an orally administered small-molecule targeted therapy, classified as a CDK4/6 inhibitor. It is primarily used in the treatment of breast cancer, particularly in the case of hormone receptor-positive, HER2-negative advanced or metastatic breast cancer^9^. Early studies suggest that the drug's mechanism of action, targeting the cell cycle, could have relevance in slowing the progression of pancreatic cancer cells^10^.

This article aims to explore the effects of Trametinib on pancreatic cancer, the mechanism of resistance formation in Trametinib-resistant cells, and the reversal of resistance strategies.

## Results

### Database screening revealed exhibits of inhibitory effects on PDAC

To identify drugs sensitive to PDAC, a thorough analysis and screening of various existing therapeutic drugs for pancreatic cancer was conducted. Information from databases such as GDSC (Genomics of Drug Sensitivity in Cancer) and CTD2 (Cancer Target Discovery and Development) was utilized in this process. A total of 297 drugs, including commonly used clinical medications like Gemcitabine, erlotinib, and sunitinib, were included in the analysis. The results of the analysis revealed that Trametinib demonstrates a significant inhibitory effect on PDAC (Supplemental Fig. 1).

### Trametinib demonstrates a superior inhibitory effect on PDAC cells compared to Gemcitabine

**Figure 1:**
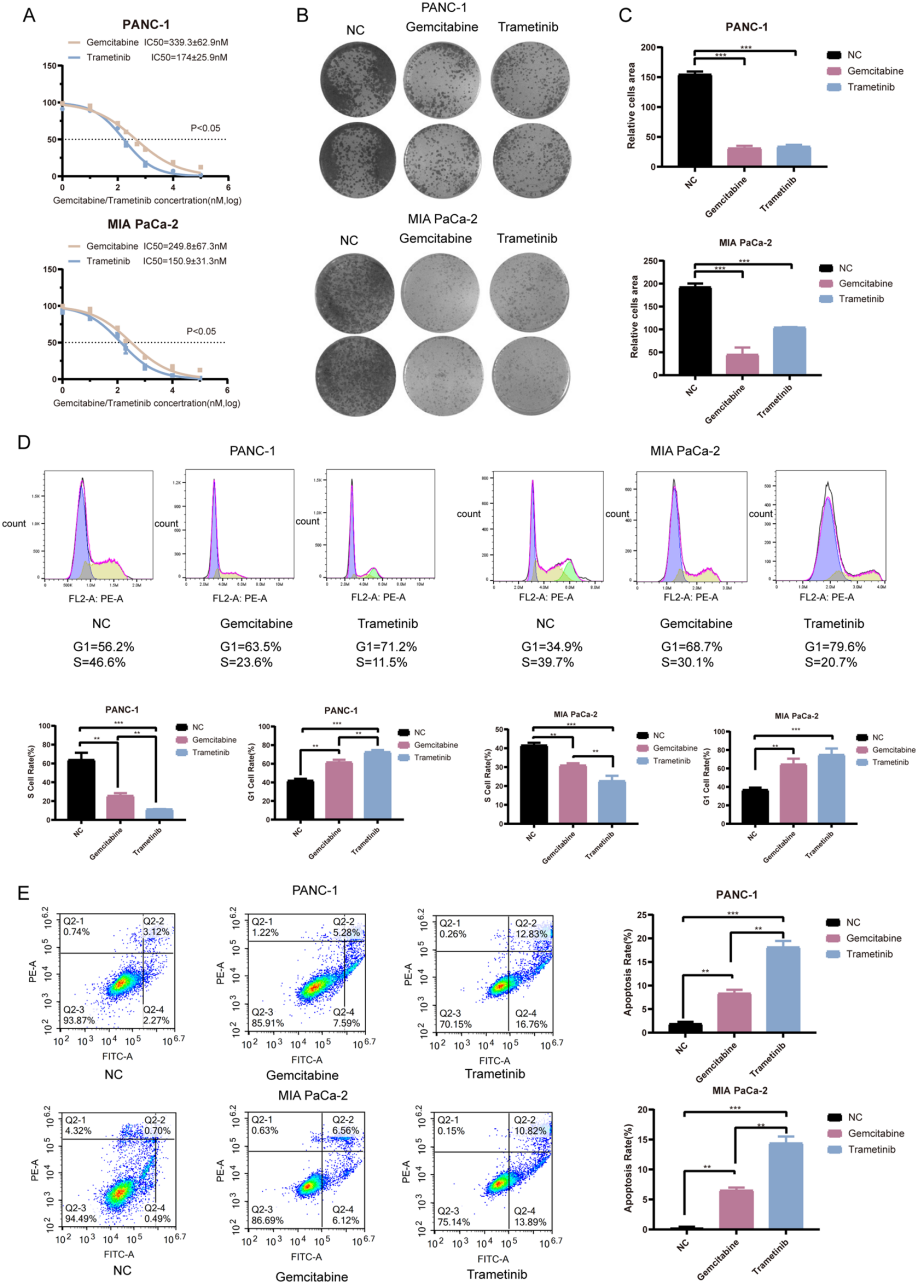
Effects of Gemcitabine and Trametinib on proliferation, cell cycle and apoptosis of PDAC cells. (**A**) CCK-8 for the IC_50_ of Gemcitabine and Trametinib in PDAC cells. (**B**) Colony formation assay of Gemcitabine and Trametinib in PDAC cells. (**C**) Data analysis of colony formation. (**D**) Cell cycle analysis of Gemcitabine and Trametinib in PDAC cells and data analysis. (**E**) Apoptosis analysis of Gemcitabine and Trametinib in PDAC cells and data analysis. Q2-2 represents late-stage apoptosis, while Q2-4 represents early-stage apoptosis. The experiments mentioned above were all repeated three times. 200 nM was selected as the standardized treatment concentration of Gemcitabine and Trametinib. *P < 0.05, **P < 0.01, ***P < 0.001.

To validate the inhibitory effect of Trametinib on the proliferation ability of PDAC cell lines, a proliferation assay was conducted using the CCK-8 (Cell Counting Kit-8) method. The results showed Trametinib had a lower IC_50_ (Half-Maximal Inhibitory Concentration) than Gemcitabine (Fig. 1A). Based on the IC_50_ concentrations of Trametinib and Gemcitabine, which range from 150 to 340 nM, the average value of 200 nM was selected as the standardized treatment concentration. Colony formation assay showed that the inhibitory effect of Trametinib on the proliferation activity of PANC-1 and MIA PaCa-2 cells was similar to the Gemcitabine group (Fig. 1B,C).

### Trametinib inhibits the cell cycle of PDAC cells

To gain further insights into the inhibitory mechanism of Trametinib on the proliferation ability of PDAC cells, cell cycle analysis was conducted. Compared to the NC group, the PANC-1 cell line showed a reduction in the S-phase cell ratio and an increase in the G1-phase cell ratio (Fig. 1D). Similar results were observed in the MIA PaCa-2 cell line (Fig. 1D). Besides, Gemcitabine showed a similar effect to the cell cycle with less inhibitory effect.

### Trametinib induces apoptosis in PDAC cells

Compared to the NC group, the Trametinib group demonstrated a significant increase in the apoptosis rate in PANC-1 cells and MIA PaCa-2 cells (Fig. 1E). Furthermore, in the comparison between the Trametinib group and the Gemcitabine group, PANC-1 cells exhibited an increase in the apoptosis rate. The similar results were shown in MIA PaCa-2 cells (Fig. 1E).

### Trametinib inhibits the downstream signaling pathways of RAS

**Figure 2:**
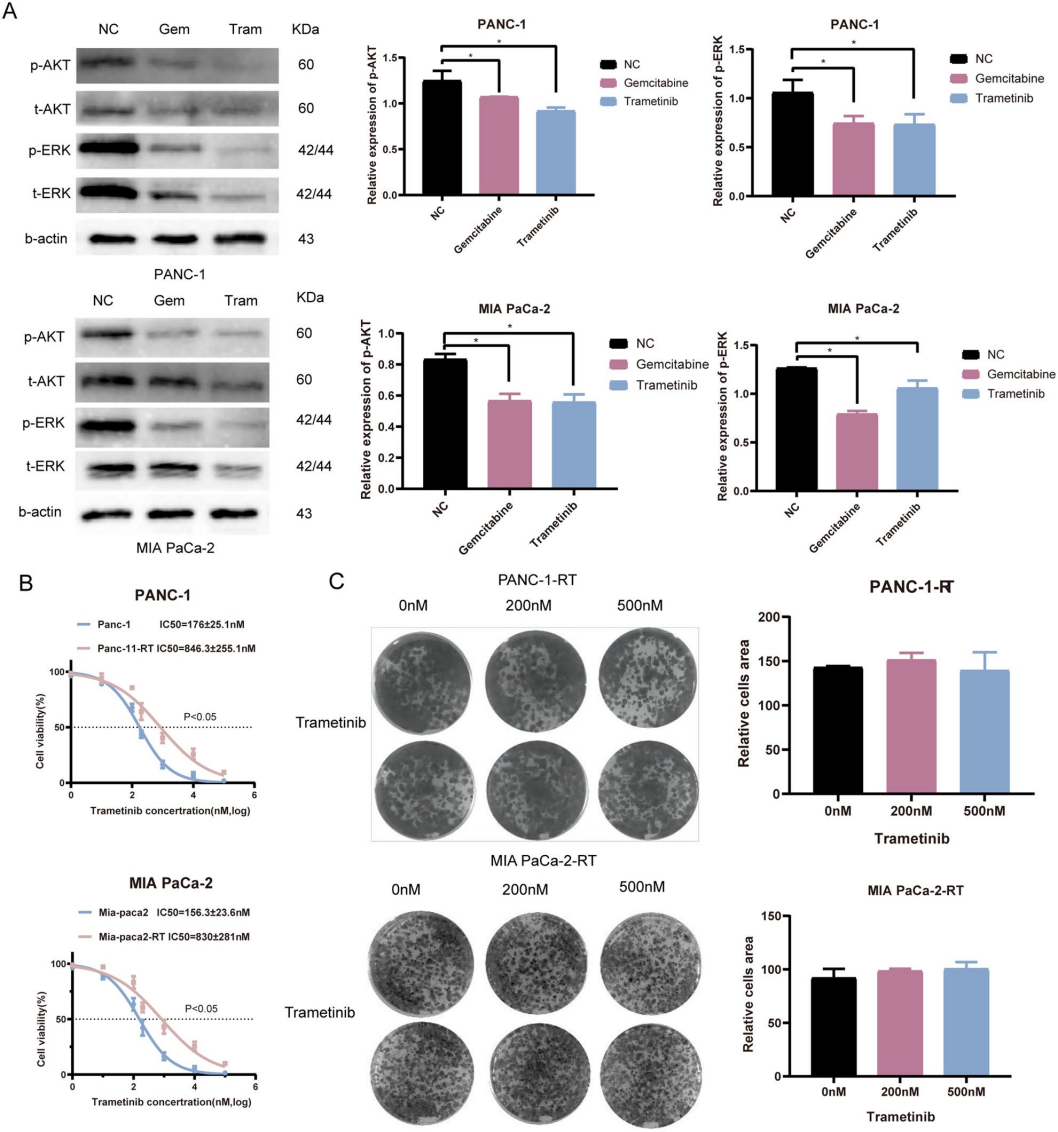
Effects of Gemcitabine and Trametinib on RAS pathway and the construction of the Trametinib resistance PDAC cell lines. (**A**) Trametinib on regulating the cell cycle and downstream signaling pathways of RAS in PDAC cells. (Original images of full-length blots cannot be provided because we often cut the blots prior to hybridization with antibodies and perform exposure). (**B**) CCK-8 tested the construction of the Trametinib resistance PDAC cell lines. (**C**) Colony assay revealed the Trametinib-resistant cell lines. The experiments mentioned above were all repeated three times. * P < 0.05, **P < 0.01, ***P < 0.001. Original blots are presented in Supplementary Fig. 2. The samples were derived from the same experiment and the gels were processed in parallel.

To investigate the molecular mechanism and effects of Trametinib on downstream signaling pathways of RAS in PDAC cells, Western blot analysis was performed. Compared to the NC group, the Trametinib and Gemcitabine group exhibited a decrease in the expression of MAPK signaling pathway-related proteins MEK I/II and PI3K/AKT signaling pathway-related protein p-ERK in both PANC-1 and MIA PaCa-2 cells (Fig. 2A). Furthermore, compared to the NC group, the Trametinib and Gemcitabine group showed a significant reduction in the expression of p-AKT proteins (Fig. 2A).

### Construction of the Trametinib resistance PDAC cell lines

Trametinib-resistant PDAC cell lines were established using the methods described in the method part. The treatment groups included PANC-1 and MIA PaCa-2 cells, as well as their corresponding Trametinib-resistant cell lines, PANC-1-RT and MIA PaCa-2-RT. Cell proliferation was evaluated using the CCK-8 assay (Fig. 2B). The colony formation assay revealed that the Trametinib-resistant cell lines demonstrated similar proliferation capability in different Trametinib concentrations (Fig. 2C).

### RNA-seq to find the mechanism of drug resistance

**Figure 3:**
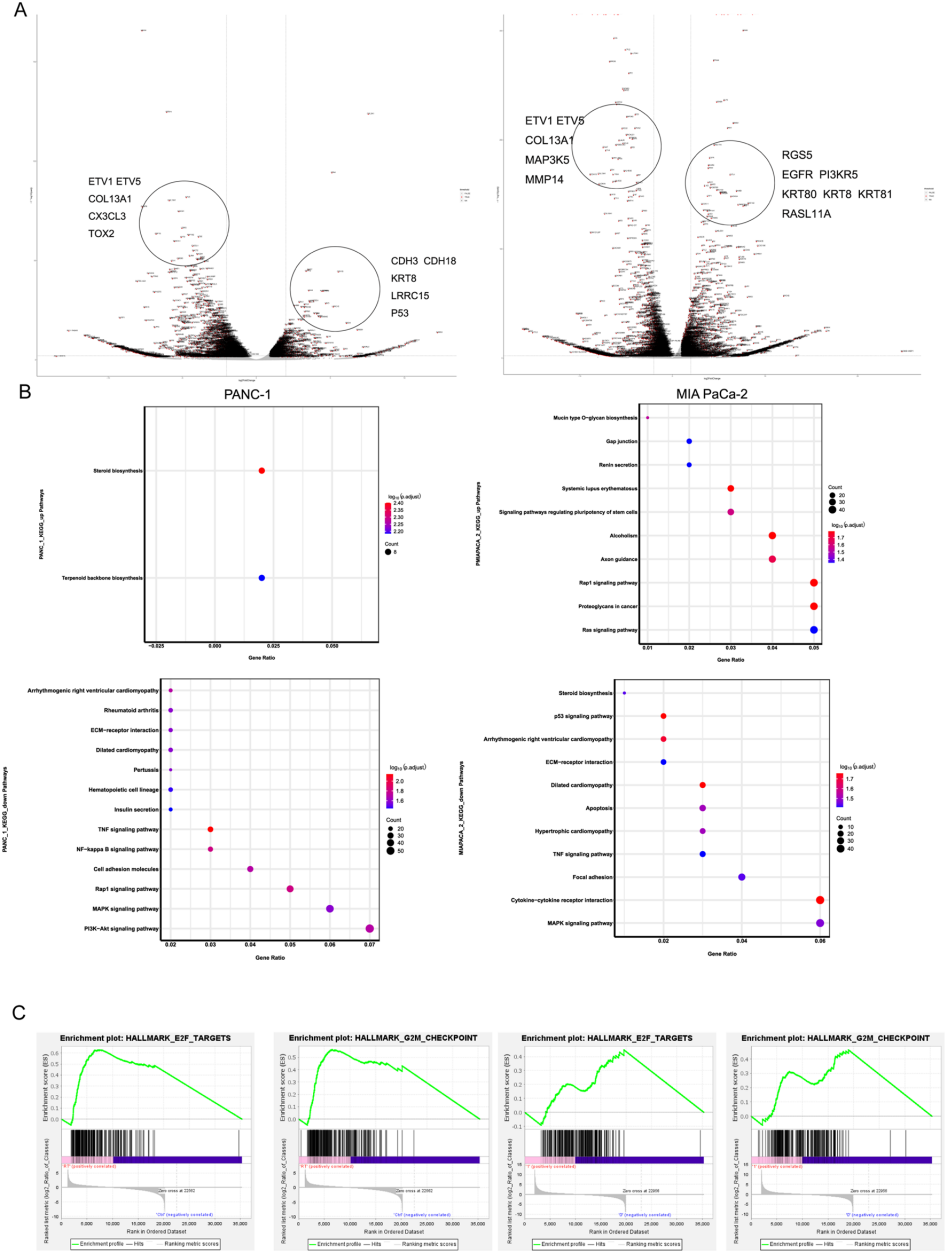
RNA-seq analysis on both normal and Trametinib-resistant cell lines. (**A**) Volcano map between normal and Trametinib-resistant cell lines. (**B**) KEGG analysis. (**C**) GSEA analysis.

To investigate the mechanisms underlying Trametinib resistance in PDAC cell lines, RNA-seq analysis was performed on both normal and Trametinib-resistant cell lines. A comparative analysis of the data revealed differentially expressed genes, in different groups. The resistant cell lines exhibited downregulation of genes such as ETV5, ETV1, COL13A1, MAPK1, and MAPK8. Conversely, in MIA PaCa-2-RT cells, upregulation of classic oncogenic genes such as EGFR, PI3KR3, and KRT81 was observed (Fig. 3A). On the other hand, in PANC-1-RT cells, significant upregulation of genes including CDH3, CDH18, and KRT8 was observed (Fig. 3A). GSEA (Gene Set Enrichment Analysis) analysis showed the expression of the E2F transcription factor family and the G2M checkpoint was significantly upregulated in both PANC-1-RT and MIA PaCa-2-RT cells (Fig. 3B,C).

### Palbociclib inhibits the proliferation and cell cycle of PDAC cells

**Figure 4:**
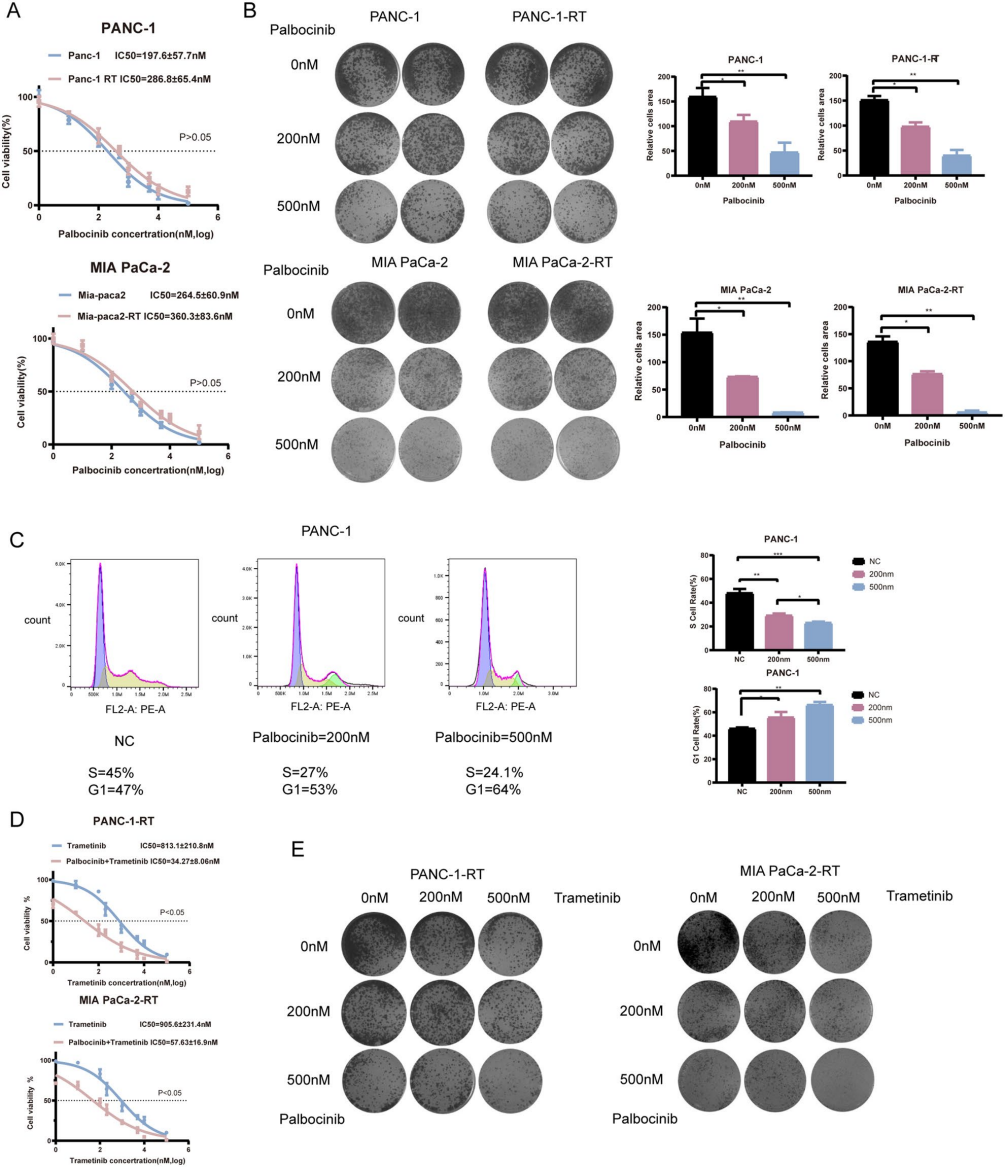
Palbocinib affects the proliferation and cell cycle of PDAC cells. (**A**) CCK-8 for the IC_50_ of Palbocinib in PDAC cells. (**B**) Colony assays for Palbocinib in PDAC cells. (**C**) Cell cycle analysis of Palbocinib in PDAC cells and data analysis. (**D**) CCK-8 for the IC_50_ of Palbocinib combined with Trametinib in PDAC cells. (**E**) Colony assays for Palbocinib combined with Trametinib in PDAC cells. The experiments mentioned above were all repeated three times. 200 nM was selected as the standardized treatment concentration of Palbocinib and Trametinib. *P < 0.05, **P < 0.01, ***P < 0.001.

Palbociclib is an inhibitor that targets the cyclin-dependent kinases CDK4/6, which are involved in the regulation of the cell cycle. CCK-8 assay was performed to validate the inhibitory effect of Palbociclib on the proliferation capability of pancreatic cancer cell lines. The results indicated that Palbociclib exhibits a stronger inhibitory effect on the proliferation activity of normal pancreatic cancer cells compared to drug-resistant pancreatic cancer cell lines (Fig. 4A). Colony assays showed similar results (Fig. 4B). In addition, the changes in cell cycle distribution of the drug-resistant cell lines under different concentrations of Palbociclib were assessed using flow cytometry analysis (Fig. 4C).

### The combined use of Trametinib and Palbociclib inhibits the proliferation of Trametinib-resistant cells

The combination treatment of Trametinib and Palbociclib on the proliferation capability of pancreatic cancer cells was validated by the CCK-8 assay. In both resistant and non-resistant cell lines, it was observed that the combination treatment of Trametinib and Palbociclib exerted a significantly stronger inhibitory effect on the cell proliferation activity compared to the individual treatments of Trametinib or Palbociclib alone (Fig. 4D).

Besides, a colony formation assay was utilized to examine the inhibitory effects of both Trametinib and Palbociclib on pancreatic cancer cells. The combination of Trametinib and Palbociclib demonstrated superior inhibitory effects on the proliferation activity of PANC-1 and MIA PaCa-2 cells compared to the individual Trametinib or Palbociclib alone (Fig. 4E).

**Figure 5:**
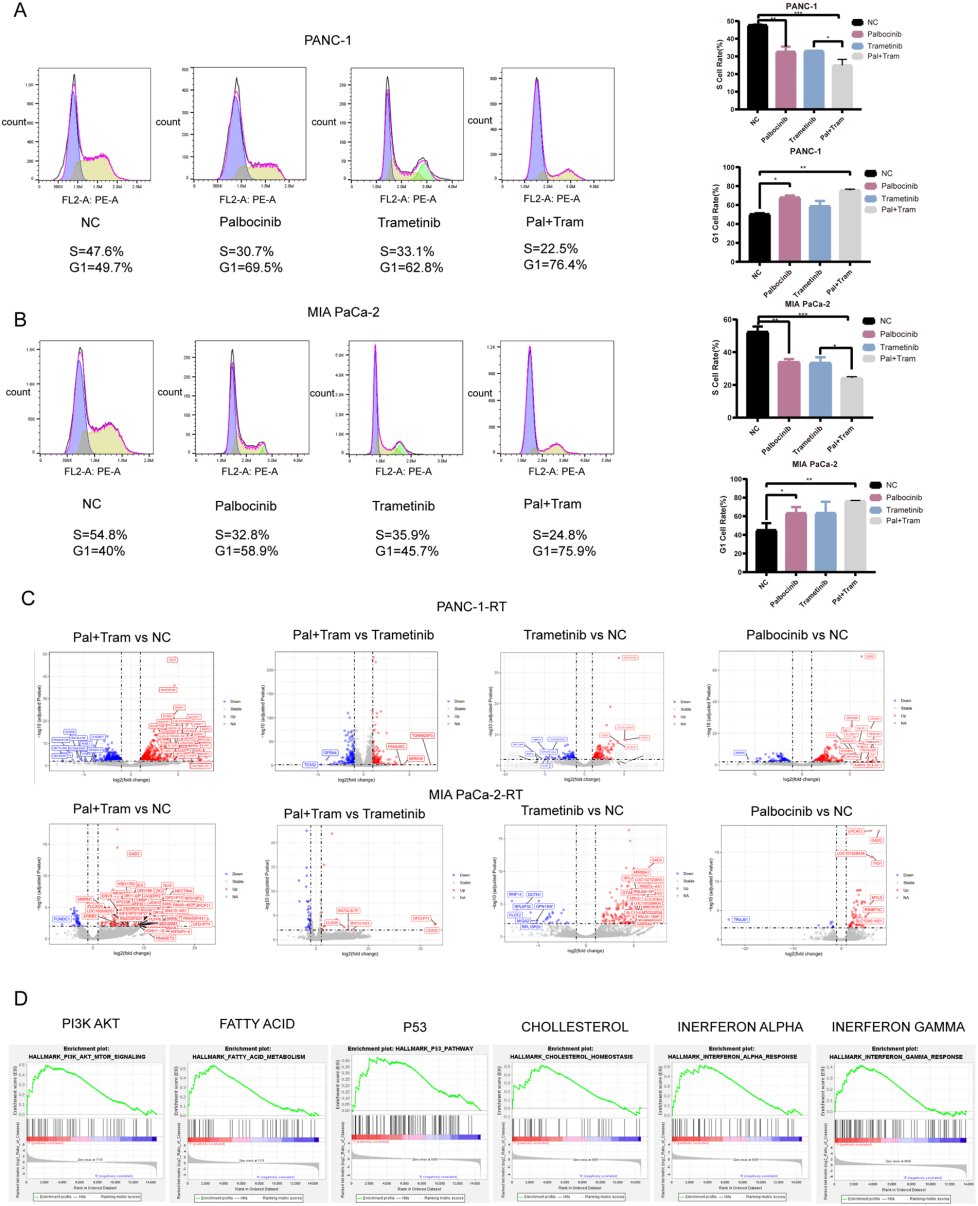
Palbocinib affects the cell cycle of PDAC cells and RNA-seq analysis to find the mechanism of the combination of Trametinib and Palbociclib. (**A**) Cell cycle analysis of Palbocinib combined with Trametinib in PANC-1. (**B**) Cell cycle analysis of Palbocinib combined with Trametinib in MIA PaCa-2. (**C**) Volcano map between normal and drug-treated cell lines. (**D**) GSEA analysis between normal and drug-treated cell lines.

Cell cycle detection analysis revealed the combination treatment of Trametinib and Palbociclib exerted a significantly stronger effect on cell cycle inhibition compared to the individual treatments (Fig. 5A,B).

### RNA-seq to find the mechanism of combination of Trametinib and Palbociclib

The study revealed that the combination of Trametinib and Palbociclib could restore the sensitivity of the resistant cell lines, PANC-1-RT and MIA PaCa-2-RT, to Trametinib. However, the specific mechanism underlying is still being investigated. To explore this further, PANC-1-RT and MIA PaCa-2-RT cell lines were divided into four groups: NC group, Trametinib group, Palbociclib group, and the combination group of Trametinib and Palbociclib.

In both PANC-1-RT and MIA PaCa-2-RT cells, over five types of KRT family genes were found to be upregulated to varying degrees. The combination treatment significantly suppressed the expression levels of these oncogenic genes (Fig. 5C). The expression levels of the interferon family were significantly upregulated. Additionally, GSEA analysis revealed that the IL6-JAK-STAT pathway, KRAS signaling pathway, as well as fatty acid and cholesterol metabolism pathways exhibited an upregulation in response to the combined group compared to the NC group (Fig. 5D).

## Discussion

Pancreatic Ductal Adenocarcinoma (PDAC), exhibits a high degree of invasiveness and resistance, leading to chemotherapy regimens^11^. There is a pressing clinical need for innovative treatment approaches to improve both survival and the quality of life for PDAC patients. In 90% of PDAC cases, the driving force is mutationally activated KRAS^12^. KRAS contributes to the progression of PDAC through various mechanisms, including malignant transformation, tumor maintenance, metabolic reprogramming, metastasis, stromal proliferation, and dysregulation of immune cells^13^. However, therapies targeting these signaling pathways have not yielded improvements in overall survival, progression-free survival, overall response rate, or response duration in untreated metastatic PDAC patients. Although mutant KRAS inhibitors are currently in development, they remain in the preclinical and early clinical research phases^14^.

The MAPK pathway has the ability to circumvent the inhibition of other signaling pathways, such as PI3K and AKT, by triggering feedback activation when these pathways are suppressed. This feedback activation often renders targeted therapies less effective. Trametinib, an oral allosteric inhibitor of MEK1/2, shows substantial promise in cancer treatment, especially in the case of tumors with mutations that activate the MAPK pathway. The RAS-RAF-MEK-ERK signaling cascade has been proven to be critical in initiating and promoting the growth of PDAC that carries activating RAF or RAS mutations^15,16^. Results from a Phase Ib combination study of Trametinib and Gemcitabine in pancreatic cancer have demonstrated the clinical effectiveness of Trametinib, providing strong support for further evaluation of this combination therapy^15,17^. Therefore, the MEK1/2 inhibitor Trametinib holds significant potential in the treatment of PDAC^18–20^. Our research findings suggest that Trametinib, through selective inhibition of MEK1/2, can significantly suppress pancreatic cancer cell proliferation and effectively halt the cell cycle progression, thus inducing cell death. Colony and cell flow found Gemcitabine and trametibine can inhibit PDAC cell proliferation. Expression levels of two proteins, ERK in the MAPK pathway and AKT in the PI3K/AKT pathway were assessed through Western Blot experiments. Both of these pathways are vital downstream signaling cascades of KRAS, and this approach serves to validate the inhibitory effects of Trametinib on KRAS downstream targets^18,21^.

The development of drug resistance to Trametinib has limited its application in the clinic. Hence, the investigation of the mechanisms behind Trametinib resistance and the establishment of Trametinib-resistant cell lines are of utmost importance. We induced drug resistance by subjecting cells to high-dose Trametinib, maintaining resistance during intervals with lower doses of Trametinib. The concentration was gradually increased until cells could consistently grow in the presence of 10 μM Trametinib, a process that spanned 6 months. The success of this resistance induction was confirmed through CCK-8 proliferation assays.

To explore the role of abnormally expressed genes in PDAC drug resistance, we employed RNA-seq and conducted KEGG enrichment analysis to identify gene sets associated with resistance. This enabled us to delve into their functions in the onset and progression of cancer. In drug-resistant cells, the expression levels of signaling pathways such as MAPK, TNF, and P53 have all shown downregulation^22^. This indicates that drug-resistant cell lines are capable of reducing their reliance on specific signaling pathways through bypass feedback mechanisms, consequently reducing the inhibitory effects of Trametinib. Additionally, in drug-resistant cell lines, there is an upregulation of cell cycle-related signaling pathways and gene expression. A noteworthy observation is the upregulation of E2F transcription factors and genes associated with the G2/M DNA damage checkpoint pathway^23–25^.

Co-administration of Trametinib with Palbociclib has the remarkable effect of restoring sensitivity to Trametinib in the drug-resistant cell lines, PANC1-RT and MIA PaCa-2-RT. The results from CCK-8 cell proliferation assays demonstrate that the combination of Trametinib and Palbociclib exhibits a significantly stronger inhibitory effect on the viability of PDAC drug-resistant cell lines compared to Trametinib monotherapy. These findings are further supported by phenotypic experiments.

A study found that MEK inhibitor in combination with CDK4/6 inhibitor has significant anti-KRAS-mutant NSCLC (Non-Small Cell Lung Cancer) activity and radiosensitizing effect in preclinical models^26^. Besides, Combined inhibition of both MEK and CDK4/6 is effective in preclinical models of KRAS mutant CRC (Colorectal Cancer) and justifies a planned phase II clinical trial in patients with refractory KRAS-mutant CRC^27^.

In PDAC, a previous study found that inhibition of MAPK (RAF > MEK1/2 > ERK1/2) signaling with Trametinib, combined with Gemcitabine, failed to improve overall or progression-free survival, overall response rate, or duration of response in untreated metastatic PDAC patients^28^. Interestingly, combined inhibition of MEK1/2(with Trametinib [T]) plus autophagy (with chloroquine [CQ] or hydroxychloroquine [HCQ]) demonstrated striking anti-tumor effects in preclinical models and in a patient. Besides, a CDK4/6 inhibitor, Palbociclib, also induced autophagy and overrode c-MYC mediated T/HCQ resistance, such that P/HCQ promoted regression of T/HCQ-resistant PDAC tumors with elevated c-MYC expression. Finally, P/HCQ treatment of patients resulted in a biochemical disease response^29^.

The result indicates the potential application of the combination of Trametinib and Palbociclib in pancreatic cancer.

Moreover, we conducted pathway enrichment analysis using GSEA based on the TCGA database to analyze the differentially expressed genomes of PANC1-RT and MIA PaCa-2-RT cells after treatment with various drug combinations, with a focus on KEGG (Kyoto Encyclopedia of Genes and Genomes) pathways. This analysis provided insights into the mechanisms responsible for the reversal of drug resistance in combination therapy.

The KEGG analysis revealed that in PANC1-RT cells, the highly expressed RNAs in the combination therapy group of drug-resistant cell lines are primarily associated with the regulation of factors such as interferon-α and interferon-γ^13,30^. The expression levels of interferon family genes are significantly upregulated in the combination therapy group cells.

There were some limitations in our study. Firstly, it relied predominantly on in vitro experiments using cell lines, which may not entirely capture the intricacies of real clinical scenarios. To substantiate our findings, further research employing patient-derived xenografts or organoids is warranted. Secondly, while we observed correlations between Trametinib and PDAC, the specific molecular mechanisms directly underpinning these associations remain unclear. Future studies that delve into the molecular pathways and signaling cascades involved in Trametinib would contribute to a more comprehensive understanding of its role in the progression of pancreatic cancer.

## Methods

### Cell lines and construction of drug resistance cell lines

The PANC-1 and MIA PaCa-2 cell lines were obtained from the Cell Bank of the Chinese Academy of Sciences. These cells were cultured in DMEM medium (Gibco, America) supplemented with 10% fetal bovine serum (Gibco, America) and 1% penicillin/streptomycin, in a 5% CO_2_ atmosphere at 37 °C. Trametinib-resistant cells were maintained under similar conditions, with the addition of 10 μM Trametinib.

The drugs, including Trametinib (Selleck, S2673, America), Gemcitabine (Selleck, S1714, America), and Palbociclib (Selleck, S1116, America) were dissolved in DMSO (Dimethyl sulfoxide). The final concentration of DMSO in all drugs was less than 0.1% in the medium to reduce cytotoxicity. To establish Trametinib-resistant cell lines, PANC-1 and MIA PaCa-2 cells were cultured in DMEM complete medium supplemented with a low concentration of Trametinib. The Trametinib concentration in the medium was gradually increased in a slow gradient, accompanied by high-dose shock induction, until pancreatic cancer cells were able to grow steadily in a medium containing 10 μM Trametinib. Changes in cell viability were assessed using the CCK-8 cell viability assay kit to confirm the development of drug resistance. The induced Trametinib-resistant cells were designated as MIA PaCa-2-RT and PANC-1-RT cells, respectively.

### Drugs IC_50_ assay by CCK-8

A total of 5 × 10^3^ cells were seeded in each well of 96-well plates under the conditions mentioned in the cell culture section. Doses of 0 nM, 10 nM, 100 nM, 1000 nM, 10 μM, and 100 μM of drugs were added to the respective wells, and the cell cultures were maintained for 24 h at 37 °C. Following the incubation period, 10 μL of CCK-8 solution mixed with 90 μL of RPMI 1640 medium was added to each well and incubated for 2 h at 37 °C before detection. A microplate reader (Thermo, Multiskan SkyHigh, America) was used to detect the OD (optical density) at 450 nm.

### Western blotting

Protein samples were separated by SDS-PAGE (Sodium dodecyl sulfate–polyacrylamide gel electrophoresis) on 10% gels, transferred onto nitrocellulose membranes, and blocked for 1 h at room temperature using Tris-buffered saline containing 0.1% Tween and 5% fat-free milk. The membranes were then probed with primary antibodies overnight at 4 °C. Subsequently, the membranes were incubated at 37 °C for 1 h with secondary antibodies, and an enhanced chemiluminescence reagent (Sigma, America) was used to visualize the bands. The bands were detected using the Chemi Doc XRS instrument with Image Lab Software (Bio-Rad, America).

The following antibodies were used: β-actin (Proteintech, 81115–1-RR, China), ERK (Zenbio, 343830, China), p-ERK (Zenbio, 310065, China), p-AKT (Zenbio, 381555, China), AKT (Zenbio, 342529, China), and HRP-conjugated rabbit-to-mouse secondary antibody (Proteintech, SA00001-1, China).

### Colony formation assay

Cells were seeded in 6-well plates at a density of 3000 cells per well and cultured for 1–2 weeks. Following the incubation period, the cells were fixed with 4% paraformaldehyde, and 0.5% crystal violet solution was used to stain and visualize the colonies.

### Cell cycle detection

The cell cycle was analyzed using an APC BrdU Flow Kit (Beyotime, China). After synchronizing the cell cycle for 24 h, the cells were resuspended and fixed with 75% ethanol for a minimum of 4 h. Subsequently, the cells were stained with propidium iodide (PI), a dye used to detect the cell cycle population by flow cytometer (BD, LSR II, America). Flowjo X software was used for data analysis.

### Cell apoptosis detection

The percentage of apoptotic cells was determined using an Annexin V Apoptosis Detection Kit (Beyotime, China). When the cell confluence reached 70–80%, the cells were detached and resuspended, and then stained with 10 µL of Annexin V-APC and PI for 20 min in the dark. The percentage of apoptotic cells was measured using a BD LSR II flow cytometer, and the results were analyzed using FlowJo X software.

### RNA-seq analysis

RNA-seq analysis was conducted using wild-type and drug-resistant cell lines. Sequencing was performed on the BGISEQ-500 platform at Genechem (Shanghai, China). The resulting clean reads were mapped to reference genes using HISAT2 v2.0.5 software. Differentially expressed genes (DEGs) were identified using DESeq2, with a cutoff of an absolute fold change ≥ 1 and an adjusted P value ≤ 0.05.

### Statistical analysis

All data were expressed as mean ± standard deviation. Figures were made by Adobe Illustrator CC 2017 version 21.0. Statistical analyses were conducted using GraphPad Prism version 9.0 and SPSS 13. For comparisons between two groups, an independent sample t-test or paired t-test was performed. One-way analysis of variance (ANOVA) followed by post hoc Tukey's test was used to compare differences among multiple groups. A significance level of P < 0.05 was considered statistically significant.

### Ethics statement

No animal or human tissue are used in the current study. The study was conducted in accordance with ethical guidelines and approved by the Ethical Committee of the West China Hospital, Sichuan University (approval number: 2019955). All experimental procedures and subjects were conducted following the guidelines and regulations of Sichuan University.

## Conclusion

In summary, we evaluated a novel combination of MEK and CDK4/6 inhibitors in PDAC cells to inhibit cell cycle and proliferation. It induced G1/S cell cycle arrest and apoptosis, presenting a notably superior treatment outcome compared to Gemcitabine. Besides, the combination of Trametinib with the cell cycle inhibitor Palbociclib was found to have the potential to reverse drug resistance in PDAC.

### Supplementary Information

Supplementary Figure 1.

Supplementary Figure 2.

## Data Availability

The datasets generated and/or analysed in this study are available from the corresponding author on reasonable request.
