# Supplementary material for: CDK4/6 inhibition sensitizes MEK inhibition by inhibiting cell cycle and proliferation in pancreatic ductal adenocarcinoma
Source: Sci Rep. 2024 Apr 10;14:8389. doi: 10.1038/s41598-024-57417-z (PMC11006845; doi:10.1038/s41598-024-57417-z)
Supplement: Supplementary file 1 — Supplementary Figure 1. [file 41598_2024_57417_MOESM1_ESM.docx]

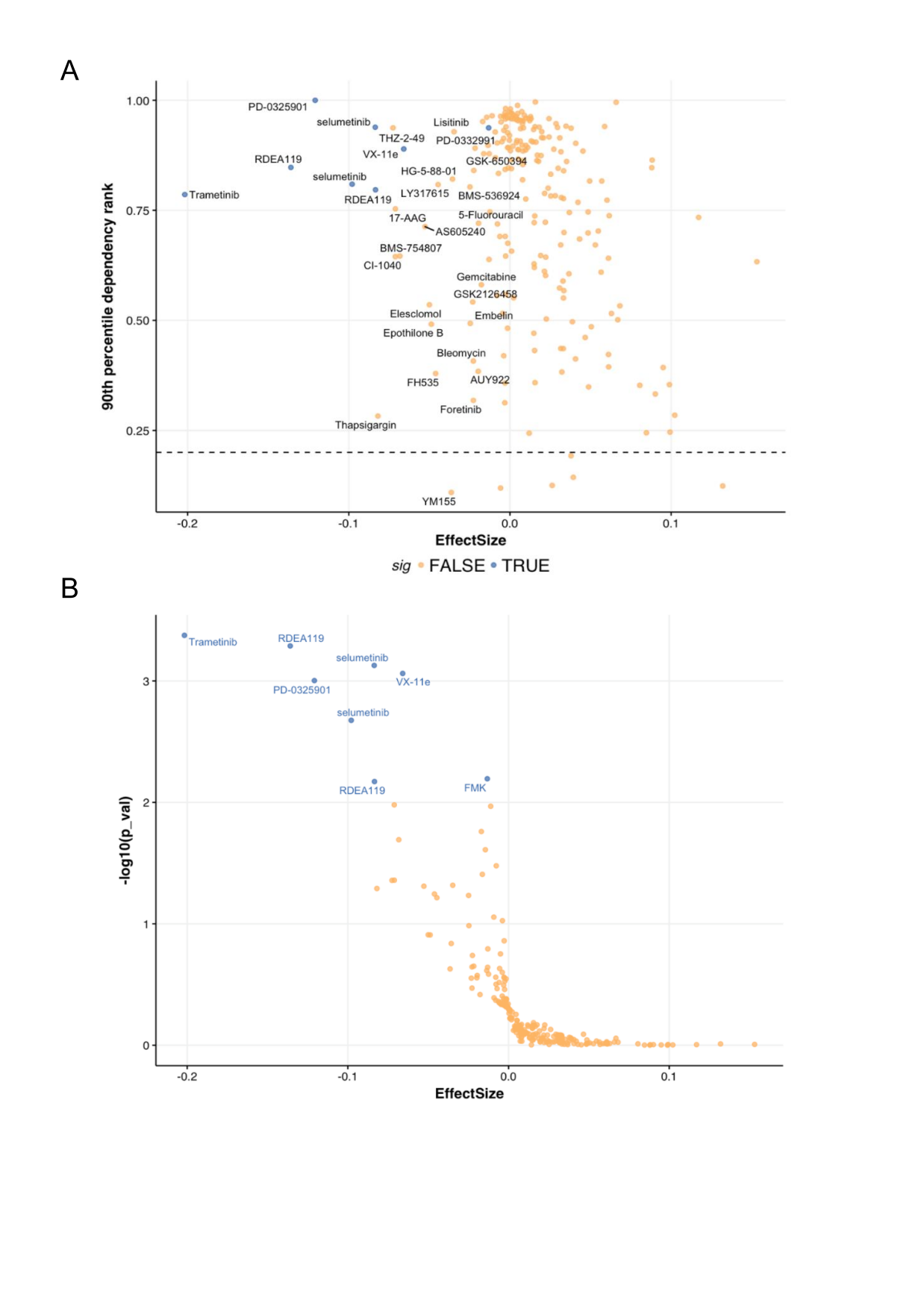


Supplement Figure 1 Pancreatic ductal adenocarcinoma (PDAC) drug sensitivity screening.

A: The tumor-suppressive effect on pancreatic cancer from GDSC (Genomics of Drug Sensitivity in Cancer)

B: The tumor-suppressive effect on pancreatic cancer from CTD2 (Cancer Target Discovery and Development)
