# Supplementary material for: CDK4/6 inhibition sensitizes MEK inhibition by inhibiting cell cycle and proliferation in pancreatic ductal adenocarcinoma
Source: Sci Rep. 2024 Apr 10;14:8389. doi: 10.1038/s41598-024-57417-z (PMC11006845; doi:10.1038/s41598-024-57417-z)
Supplement: Supplementary file 2 — Supplementary Figure 2. [file 41598_2024_57417_MOESM2_ESM.pdf]

Supplement Figure2 Origin images of western blotting

**We used red boxes to mark the regions of the original blots used in main figures and some replicates marked by black boxes.**

Cropped blots in main paper | Orginal blots in supplementary information

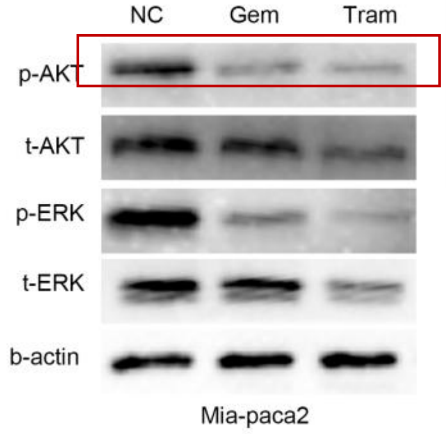


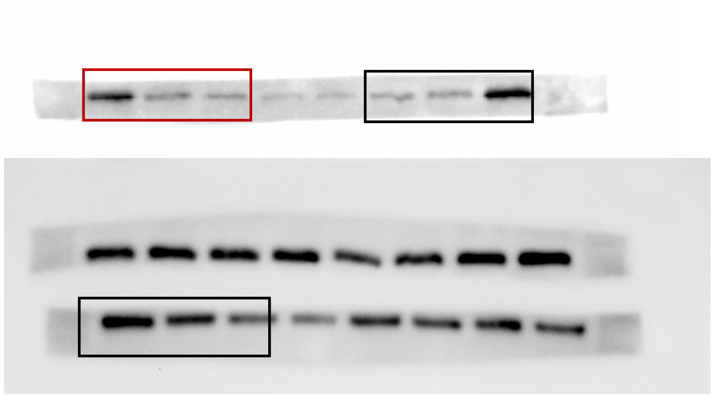

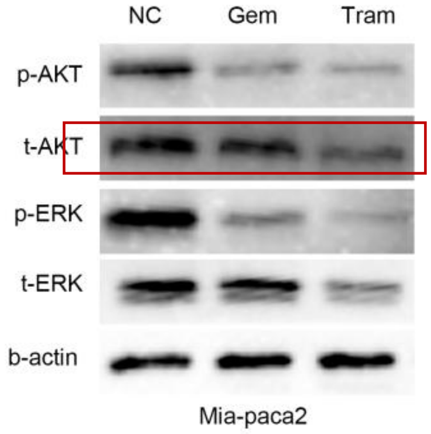


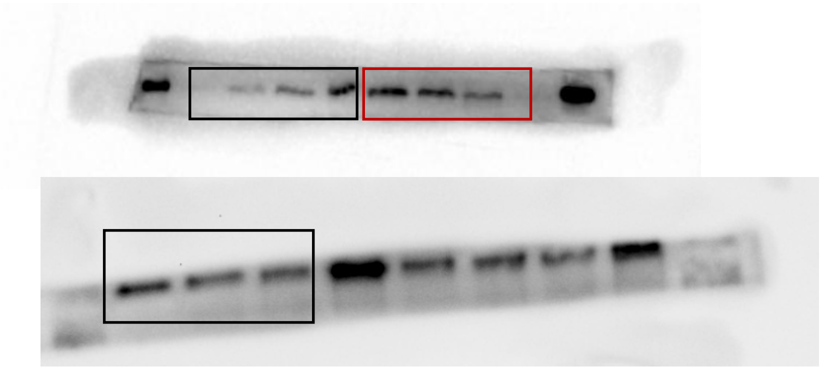

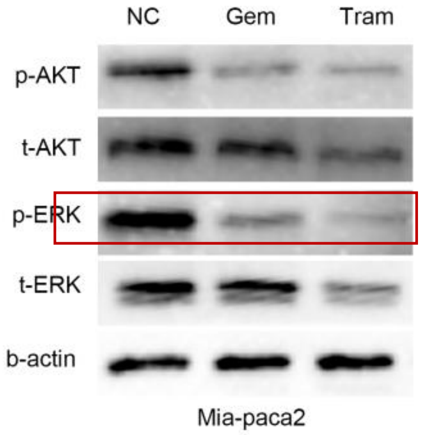


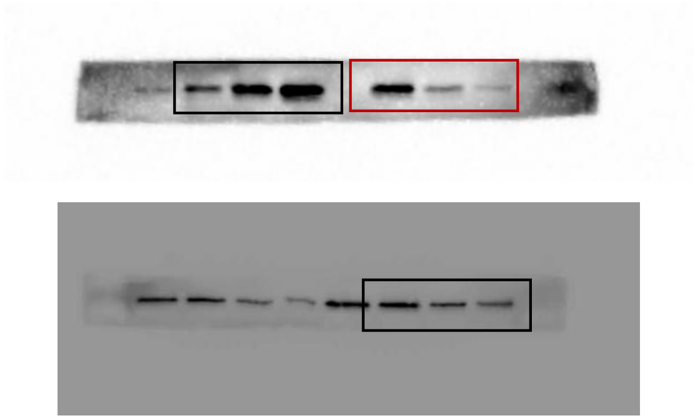

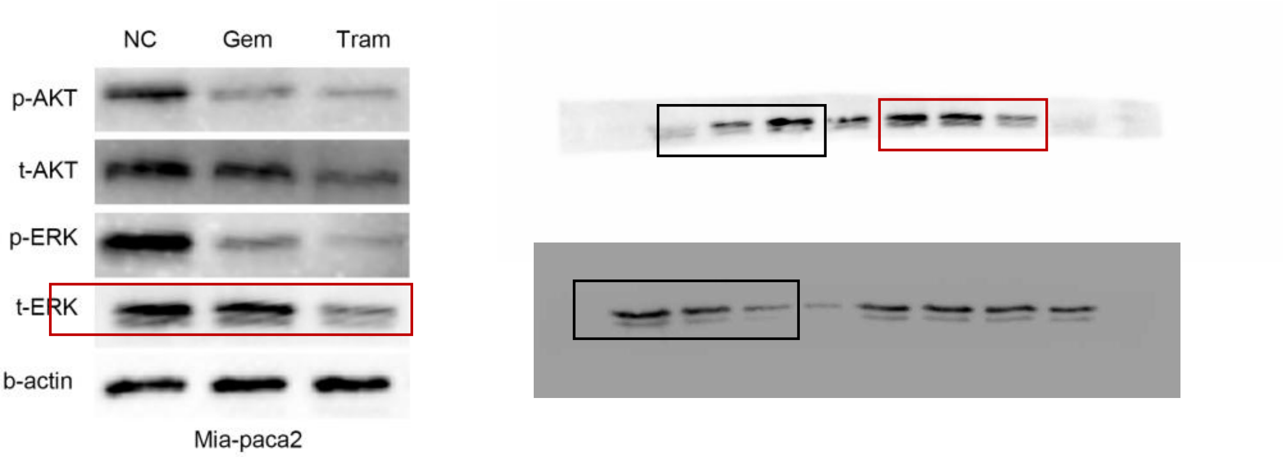
NC
Gem
Tram
p-AKT
t-AKT
p-ERK
t-ERK
b-actin
Mia-paca2

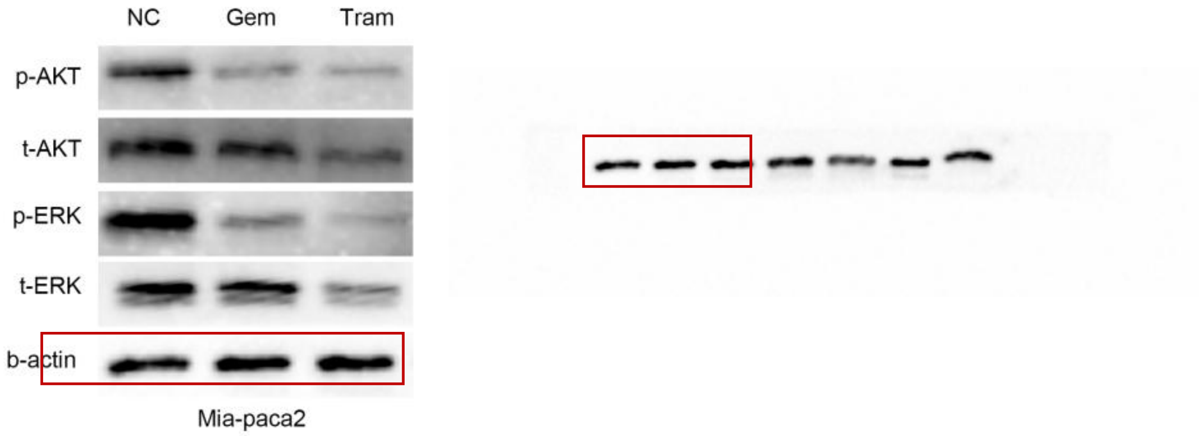
NC
Gem
Tram
p-AKT
t-AKT
p-ERK
t-ERK
b-actin
Mia-paca2

Cropped blots in main paper

Orginal blots in supplementary information

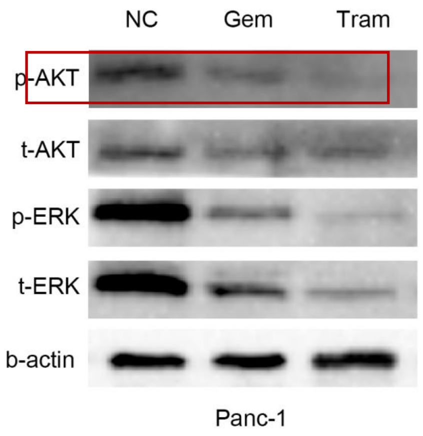


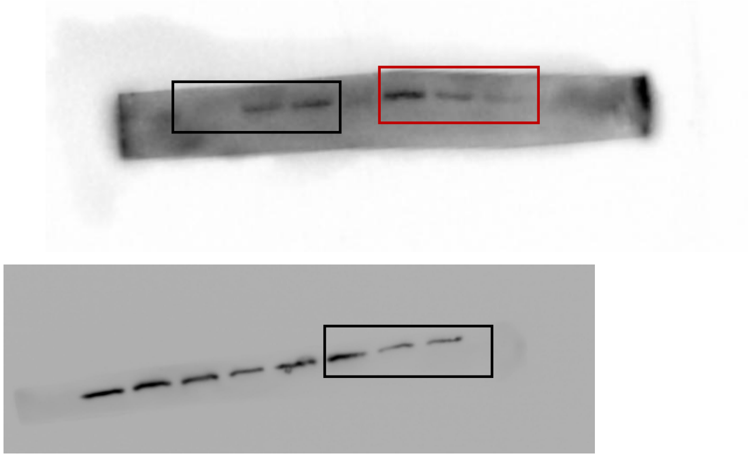

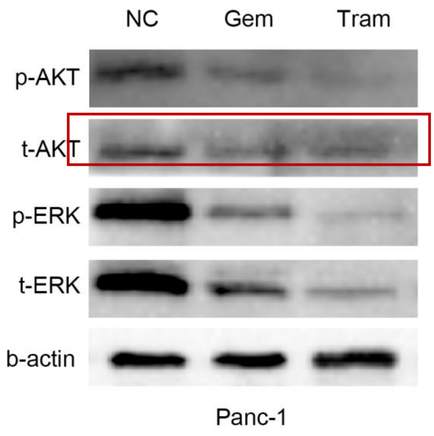


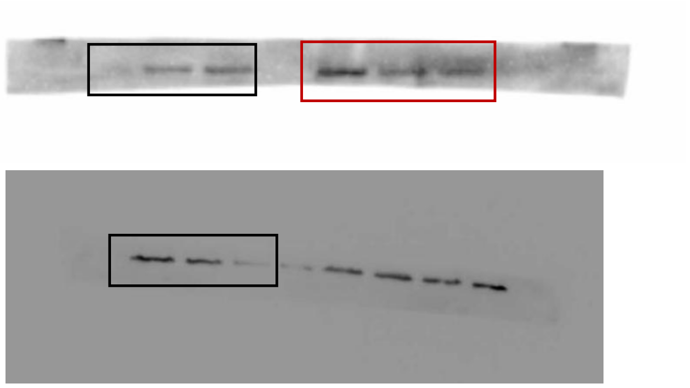

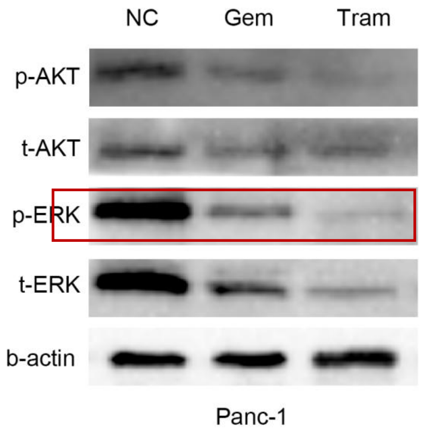


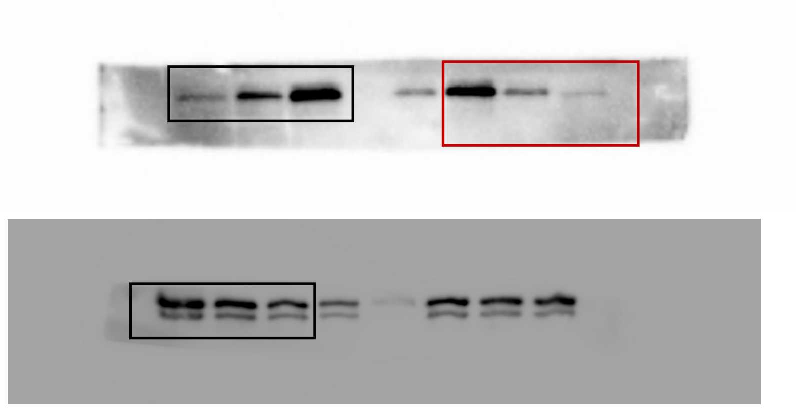

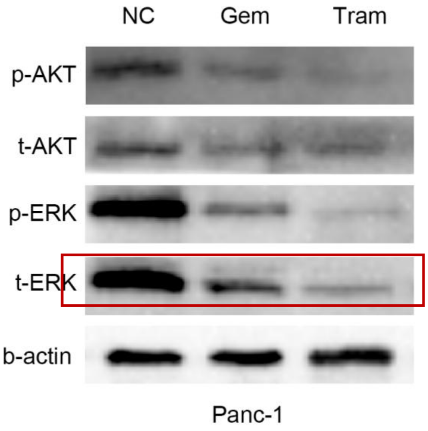
NC
Gem
Tram
p-AKT
t-AKT
p-ERK
t-ERK
b-actin
Panc-1

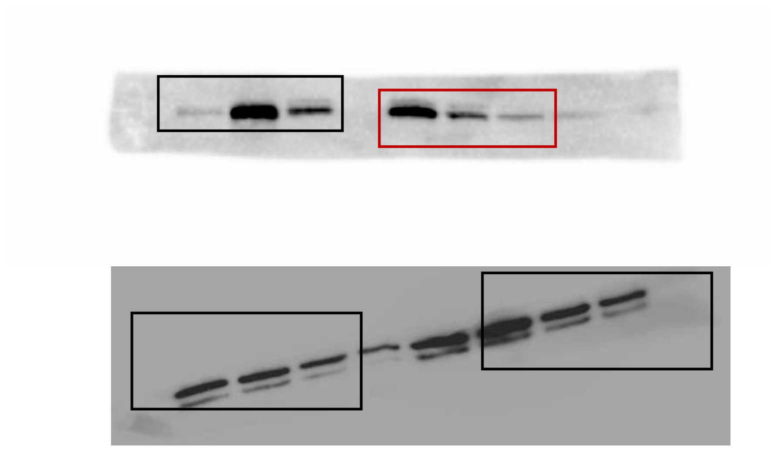

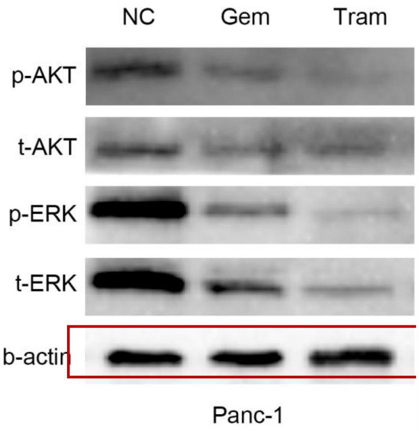
NC
Gem
Tram
p-AKT
t-AKT
p-ERK
t-ERK
b-actin
Panc-1

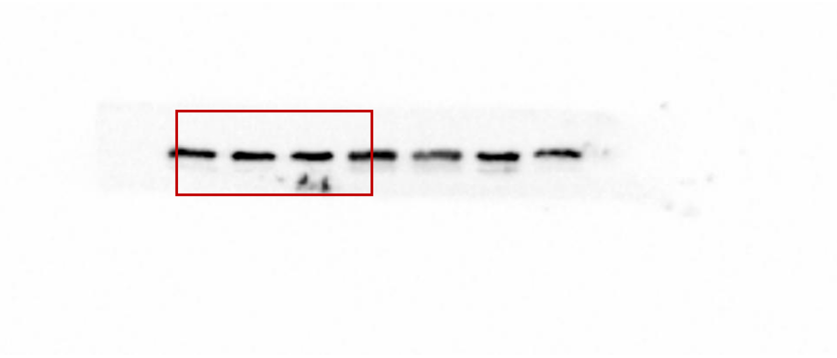

Terk

Actin
